# Management of Iatrogenic Bile-Duct Injury After Cholecystectomy, 1995–2025: Systematic Review and Meta-Analysis

**DOI:** 10.3390/life15121858

**Published:** 2025-12-03

**Authors:** Catalin Piriianu, Elena-Adelina Toma, Octavian Enciu, Mugur Ardelean, Adrian Miron, Valentin Calu

**Affiliations:** 1 Department of Surgery, Elias University Emergency Hospital, 011461 Bucharest, Romania; 2 Department of Surgery, Carol Davila University of Medicine and Pharmacy, 020021 Bucharest, Romania

**Keywords:** bile duct injury, cholecystectomy, hepaticojejunostomy, surgical timing, referral, hepatopancreatobiliary center, meta-analysis, ERCP

## Abstract

Iatrogenic bile duct injury (IBDI) constitutes a major complication of cholecystectomy. The optimal timing, method, and setting for definitive repair remain subjects of debate. This study aimed to systematically evaluate management strategies, timing of repair, and prognostic factors influencing postoperative outcomes following IBDI. A systematic review and meta-analysis were conducted in accordance with PRISMA and MOOSE guidelines (PROSPERO CRD420251003227). PubMed and the Cochrane Library were searched through March 2025. Eligible randomized trials and cohort studies reporting management outcomes were included. Data extraction and quality assessment were performed independently. Pooled analyses were conducted using random-effects models. Twenty-eight studies (2 randomized trials, 24 cohort studies, 2 systematic reviews) involving >18,000 patients were analyzed. Surgical repair achieved higher success than endoscopic therapy (92.6% vs. 76.1%; RR 1.22, 95% CI 1.10–1.35) and reduced stricture risk (RR 0.24, 95% CI 0.15–0.38). Roux-en-Y hepaticojejunostomy provided durable outcomes (success 83.5%; stricture 8.9%). Early (<2 weeks) or delayed (>6 weeks) repair after sepsis control was associated with lower morbidity (9–11%) compared with intermediate repair (2–6 weeks). Referral to hepatopancreatobiliary (HPB) centers reduced complications (RR 0.32, 95% CI 0.23–0.46). Overall morbidity and mortality were 22.7% and 2.9%. Outcomes following IBDI are determined primarily by surgical expertise and patient stability rather than timing alone. In optimized patients, both early and delayed reconstruction are safe and effective, whereas intermediate repair and non-specialist interventions increase risk. Timely referral to HPB centers should be considered standard practice.

## 1. Introduction

Iatrogenic bile duct injury (IBDI) is a significant and potentially life-altering complication that can occur after cholecystectomy, particularly in laparoscopic surgery, which is now the standard treatment for gallstone disease. Laparoscopic cholecystectomy (LC) provides expedited recovery and reduces complications; nonetheless, it is consistently associated with an elevated incidence of bile duct injury relative to open surgery [1,2,3]. Similar systematic reviews conducted by our group on colorectal and hepatobiliary surgery have emphasized the impact of surgical expertise and perioperative infection control on postoperative outcomes [4,5,6]. The incidence of IBDI following laparoscopic cholecystectomy is low, ranging from 0.1% to 1.3%. However, these injuries tend to be more complex than those associated with open surgery due to constraints in visual depth and potential anatomical misidentification [7,8,9].

The ramifications of the IBDI persisted beyond the initial trauma. Patients may experience bile leaks, biliary strictures, recurrent infections, and progressive liver damage, adversely affecting their quality of life and long-term survival [10,11,12,13,14]. Research has indicated that even “minor” injuries can result in significant morbidity, especially when diagnosis or treatment is postponed [15,16]. Bile duct injuries are a prevalent cause of medicolegal claims post-abdominal surgery and impose considerable strain on healthcare systems due to prolonged hospitalizations, many treatments, and intricate reconstructions [7,17].

Bile duct injury arises from various factors, predominantly human error, especially the inability to identify the normal anatomy inside Calot’s triangle [16,18]. Misidentification constitutes > 70% of biliary duct injuries (BDIs), even in procedures where the Critical View of Safety (CVS) is pursued [2,8]. Risk factors including acute or subacute cholecystitis, structural distortion, thicker gallbladder walls, and variations in the cystic duct anatomy markedly elevate the likelihood of injury [9,19]. Emergency surgery, female sex, and impaired liver function have been associated with an increased risk of BDI [2,3].

Mitigating these injuries continues to be a surgical imperative. Intraoperative instruments such as intraoperative cholangiography (IOC), laparoscopic ultrasonography, and near-infrared fluorescence cholangiography (NIRF-C) have been suggested to enhance visualization and reduce anatomical ambiguity [16,20]. Among these, IOC is arguably the most extensively researched. A comprehensive meta-analysis showed that routine intraoperative cholangiography considerably decreased the risk of bile duct injury compared with selective use, and was cost-effective from a healthcare system standpoint [20]. Nonetheless, its practical implementation is irregular, particularly in nonspecialized or low-volume facilities [10,19].

Injuries necessitate complex care decisions that must be customized according to the degree and type of injury, the patient’s clinical condition, and available expertise [12,15]. Minor leaks may be effectively treated with endoscopic or percutaneous methods, whereas intricate transections require reconstructive surgery, typically Roux-en-Y hepaticojejunostomy [7,8]. Vascular injuries, commonly associated with significant blunt traumatic injuries, worsen the situation and elevate the risk of anastomotic failure and long-term stricture [3,12].

The timing of the repair remains a contentious issue. Conventional perspectives advocate for postponed reconstruction, deferring the procedure for several weeks until the inflammation diminishes. Recent multicenter data support this assumption, indicating that results may be equivalent or superior to those of early repair when conducted by proficient hepatobiliary surgeons [15,17]. This indicates that surgical proficiency, rather than timing alone, may be the critical determinant of achieving favorable outcomes [8,14].

Ultimately, categorization systems are crucial in directing management; however, they differ in complexity and clinical significance. The Strasberg and Bismuth systems are still prevalent; however, contemporary frameworks such as the Stewart-Way and Hannover classifications incorporate vascular injury and damage mechanisms, thereby providing more accurate guidance [8,16]. However, no single system fulfills all clinical requirements, and additional refining or harmonization may be essential to standardize care across institutions.

This review thoroughly assessed the existing evidence regarding the care of iatrogenic bile duct injuries, emphasizing the comparative outcomes of endoscopic, percutaneous, and surgical approaches in response to these obstacles and knowledge deficiencies. It consolidates the findings of randomized trials, observational studies, and systematic reviews to elucidate the most efficacious interventions, their timing, and their effects on long-term outcomes. Our objective was to enhance decision making, refine referral processes, and facilitate the establishment of standardized treatment protocols for this intricate yet vital surgical complication.

This review presents a synthesis of comparative outcomes for surgical, endoscopic, and percutaneous management of iatrogenic bile duct injury, emphasizing the effects of repair timing, injury severity, and specialist referral. This study examines the existing gap in quantitative data regarding the influence of these factors on long-term stricture and mortality rates.

## 2. Materials and Methods

### 2.1. Study Design and Registration

This systematic review and meta-analysis followed the PRISMA 2020 [21] and MOOSE guidelines [22]. The review protocol was registered with the International Prospective Register of Systematic Reviews (PROSPERO) [23] under the registration number CRD420251003227, accessible at https://www.crd.york.ac.uk/PROSPERO/view/CRD420251003227 (accessed on 7 April 2025). Detailed PRISMA and MOOSE checklists are provided in the Supplementary Material.

### 2.2. Data Sources and Search Strategy

A systematic search was conducted in PubMed and the Cochrane Library from inception to March 2025, utilizing MeSH terms and keywords pertinent to iatrogenic bile duct injury, surgical and endoscopic management, and clinical outcomes. No restrictions on language were implemented. The reference lists of included articles were reviewed to identify additional studies. The search was limited to PubMed and the Cochrane Library, which index most hepatobiliary surgical research; however, databases such as Embase, Scopus, and Web of Science were not searched, and this limitation is acknowledged. The comprehensive search strategy is outlined in the Supplementary Material.

### 2.3. Study Selection

Randomized controlled trials, cohort studies, and systematic reviews reporting outcomes for patients with iatrogenic bile duct injury following cholecystectomy were included. Studies included in the analysis reported at least one of the following outcomes: surgical success, stricture, morbidity, or mortality. Narrative reviews, case reports, and conference abstracts were omitted from consideration. Two reviewers conducted independent screenings of all titles, abstracts, and full texts, resolving any discrepancies through consensus.

**Figure 1:**
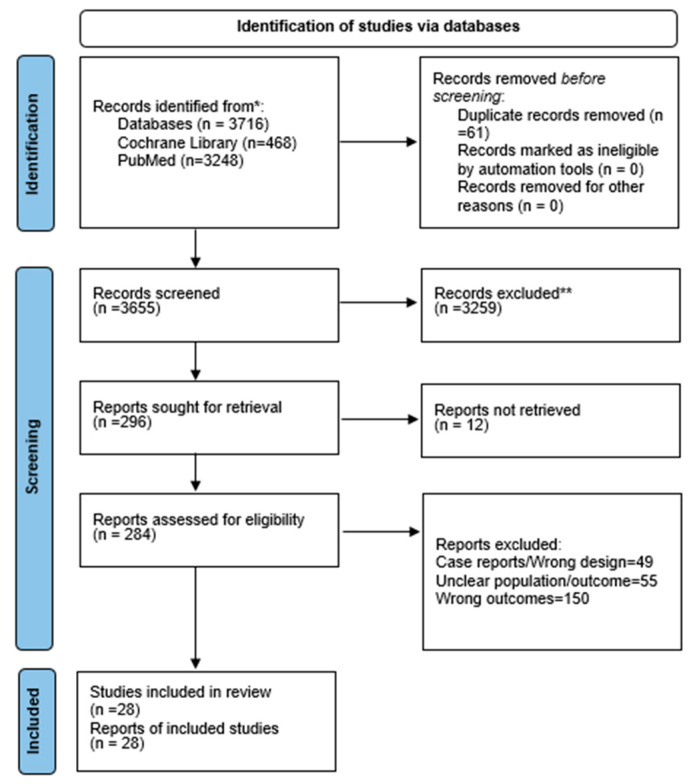
Prisma flow chart. *: Full-text articles that could not be retrieved. **: Full-text articles excluded after assessment, with reasons (e.g., inappropriate population, outcomes, or study design).

Systematic reviews were not eligible for quantitative synthesis, and no numerical data were extracted from them. Only primary studies (RCTs and cohort studies) contributed data to the pooled analyses. The PRISMA 2020 checklist and the PRISMA flow diagram (Figure 1) have been updated accordingly to reflect these corrections.

Both laparoscopic and open cholecystectomies were eligible for inclusion. ASA class and comorbidities were not used as selection criteria, as these variables were not consistently reported across studies.

### 2.4. Data Extraction and Quality Assessment

Two reviewers independently extracted data using a standardized form, which encompassed study design, setting, sample size, injury classification, intervention, timing, and outcomes. The primary outcome was the success of definitive repair, defined as a stricture-free biliary anastomosis without re-intervention. Secondary outcomes were postoperative stricture, overall morbidity, and mortality. ASA class and comorbidities were extracted when reported, but these variables were not consistently available across studies and could not be incorporated into adjusted comparisons. The severity of cholecystitis was extracted when available, but most studies did not report this variable, preventing its inclusion in comparative or pooled analyses.

The assessment of bias was conducted using the Revised Cochrane Risk of Bias tool (RoB 2) for randomized trials, the Newcastle–Ottawa Scale for cohort studies, and AMSTAR 2 for systematic reviews.

### 2.5. Statistical Analysis

Outcomes were synthesized both narratively and through meta-analysis employing random-effects models. Pooled risk ratios (RRs) and 95% confidence intervals (CIs) were computed. Heterogeneity was evaluated using the I^2^ and τ^2^ statistics. Funnel plots were utilized to assess publication bias. The certainty of evidence was assessed utilizing the GRADE methodology. Analyses were performed using RevMan version 5.4 (The Cochrane Collaboration, London, UK) and SPSS Statistics version 21.0 (IBM Corp., Armonk, NY, USA).

Risk ratios were calculated for comparative analyses, including surgical versus endoscopic management, timing groups, sepsis versus no sepsis, specialist versus non-specialist repair, and combined versus isolated bile duct injury. For outcomes without a comparator, such as overall success and stricture rates after hepaticojejunostomy, single-arm proportional meta-analyses were performed to pool event rates across studies.

For comparisons of specialist versus non-specialist repair, event counts were taken from the original studies; these numbers were not included in Table 1, which functions only as a descriptive summary.

## 3. Results

### 3.1. Search Results

PubMed and the Cochrane Library were used for a comprehensive literature review. The Cochrane Library found 468 records, whereas PubMed found 3248. The titles and abstracts of 3655 articles were reviewed after EndNote X9 had removed 61 duplicates. After screening, 296 full-text articles were assessed for eligibility. Twelve studies were found to be unrecoverable. A total of 205 papers were removed for inappropriate results or demographics, and 51 for improper study design. The final synthesis included 28 studies. The PRISMA flow diagram (Figure 1) shows the screening process.

Google Scholar was used to manually search for additional studies. The Cochrane Library and PubMed databases were used for this study. Exporting retrieved references to EndNote X9 helped to eliminate duplicates and organize citations for screening. Complex features such as MeSH explosions and automation were not used.

### 3.2. Characteristics of Included Studies

The 28 studies were conducted in various European, Asian, and North American healthcare contexts. Two randomized controlled trials [24,25], and 24 retrospective observational cohorts were included in the quantitative analysis. Two systematic reviews [14,35] were assessed narratively and were not part of the analytic dataset. Study [26] (Ma 2021) is a primary cohort study and formed part of the analytic dataset. Higher case complexity and surgical expertise have been found in most tertiary referrals and academic centers. The Strasberg classification (used in at least 16 studies) dominated the bile duct injury categorization, followed by the Bismuth and EAES versions.

Table 1 summarizes the key characteristics, management strategies, and clinical outcomes of all included studies. This encompasses study design, sample size, classification employed, type and severity of injury, management approaches (surgical, endoscopic, percutaneous), timing, primary outcomes, and the proportion of specialist HPB involvement.

All the studies examined cholecystectomy patients with iatrogenic bile duct damage. The majority (n = 22) focused on post-laparoscopic cholecystectomy injuries, whereas the rest combined open and laparoscopic surgery. Surgical reconstruction (usually Roux-en-Y hepaticojejunostomy), endoscopic management with stenting, and conservative or radiological methods were also investigated.

### 3.3. Distribution of Outcomes and Focus of Studies

The 28 studies included primarily assessed postoperative outcomes such as stricture-free survival, overall morbidity, and all-cause mortality. Surgical success, characterized by a stricture-free anastomosis without the necessity for reintervention, was documented in 24 studies. Postoperative morbidity, including cholangitis, bile leakage, wound infection, and reoperation, was reported in 22 studies. Twenty studies indicated low postoperative mortality, primarily due to sepsis or multi-organ failure. The absence of anastomotic stricture was identified as the most common predictor of long-term surgical success, as evidenced by 19 studies. The timing of repair was assessed in 16 studies, generally classified as early, intermediate, or delayed, with thresholds varying from 14 to 42 days.

Health-related quality of life, economic outcomes, and preventive intraoperative strategies—such as intraoperative cholangiography or laparoscopic ultrasonography—were infrequently assessed [27,28,29,30]. Supplementary Table S1 provides a detailed summary of the methodological characteristics and results.

### 3.4. Quality of Included Studies

The methodological quality of the studies was evaluated using validated tools. The Cochrane Risk of Bias 2 (RoB 2) assessment found a low-to-moderate bias in two randomized controlled trials [24,25]. The Newcastle–Ottawa Scale (NOS) assessed 24 observational cohort studies for study group selection, cohort comparability, and outcome ascertainment. Fifteen studies (NOS score ≥ 7) were deemed high-quality, including those by Anand et al. (2021) [27], Giuliante et al. (2023) [28], Felekouras et al. (2015) [31], and Mishra et al. (2013) [32]. Nine moderate-quality studies scored 5–6 [33,34,36].

The NOS quality score was 5 or higher in all cohort studies. The two systematic reviews were evaluated using AMSTAR 2, which is the appropriate tool for assessing the quality of systematic reviews [14,35]. Although outcome reporting is typically adequate, several observational studies have been limited by their retrospective design, potential selection bias, and inconsistent outcome definitions, particularly for surgical success and postoperative complications. Table 2 summarizes the quality assessment results for each study type.

### 3.5. Surgical Repair

Of the 28 studies reviewed, Roux-en-Y hepaticojejunostomy (HJ) emerged as the predominant reconstruction method for iatrogenic bile duct injuries, particularly in cases involving Strasberg E2–E5 lesions. T-tube repair and duct-to-duct anastomosis were designated for lower-grade injuries (Strasberg A–D) and exhibited increased complication rates. Pooled descriptive analysis indicated that HJ attained the highest success rates exceeding 90% and the lowest incidence of stricture below 6%, especially in specialized hepatobiliary centers. The findings support HJ as the optimal method for the definitive repair of significant bile duct injuries (Table 3).

#### 3.5.1. Timing of Surgical Repair

Sixteen studies assessed the influence of surgical timing on outcomes following bile duct injury repair, with primary emphasis on hepaticojejunostomy. Although definitions of “early,” “intermediate,” and “delayed” repair vary, there is agreement that timing alone does not dictate clinical success. Factors such as sepsis control, bile peritonitis, and nutritional status were the most influential. A pivotal randomized trial demonstrated that early repair following sepsis control and delayed repair produced comparable high success rates (>91%). In contrast, early intervention without sepsis control led to a lower success rate (80.9%) and the highest complication rates. Later retrospective studies established that the resolution of infection and the optimization of patient condition were more significant than the timing interval. A meta-analysis indicated a rise in morbidity associated with intermediate repairs (2–6 weeks), whereas other studies demonstrated high success rates and low stricture rates (<6%) across all timing groups. These findings indicate that optimal outcomes are more contingent upon clinical stability and specialist expertise than on rigid compliance with postoperative timing windows.

#### 3.5.2. Anastomotic Stricture and Success of Hepaticojejunostomy (HJ)

Roux-en-Y hepaticojejunostomy (HJ) is a dependable method for reconstructing major iatrogenic bile duct injuries. In 24 series, the definition of success—patent, stricture-free anastomosis without re-intervention—remained consistent, with individual success rates closely grouped between 87% and 98%. A random-effects meta-analysis of the six studies reporting full denominators indicated an overall success rate of 83.5% (95% CI 81.6–85.3%), thereby confirming the consistency of these single-center results at the aggregate level.

Late stricture represents the primary mode of failure. Upon excluding one zero-event study, the pooled incidence of stricture was determined to be 8.9% (95% CI 7.4–10.5%). The timing effects were minimal: intermediate repair (2–6 weeks) presented a 1.5-fold increased risk compared to delayed repair, whereas early repair (<2 weeks) did not demonstrate a significant difference when compared to delayed repair in two mid-sized cohorts.

Outcomes are significantly influenced by both the operator and the center involved. National registry data indicate reduced stricture and re-operation rates in tertiary hepatopancreatobiliary (HPB) units. A concurrent multicenter audit revealed a first-time success rate of 61% with HPB specialists compared to 34% with non-specialists.

In summary, HJ establishes durable biliary continuity in approximately 80% of patients overall and exceeds 90% when conducted early by skilled HPB teams; the remaining 9% risk of stricture highlights the necessity for long-term monitoring.

**Figure 2:**
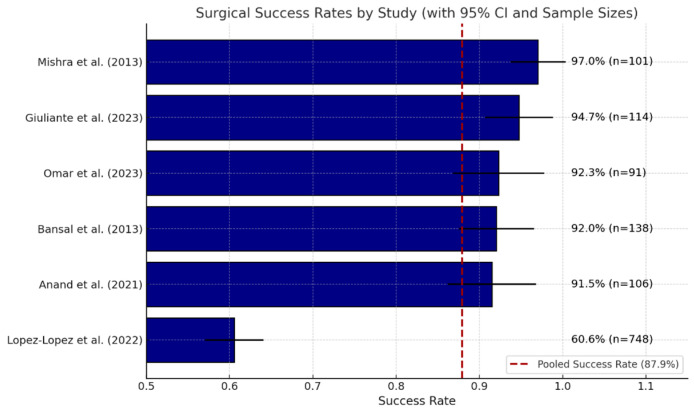
Surgical Success Rates by Study Including 95% Confidence Intervals and Sample Sizes [25,27,28,32,39,40].

Figure 2 presents a horizontal bar chart depicting the success rates of hepaticojejunostomy (HJ) procedures as reported in six studies [25,27,28,32,39,40].

Each bar illustrates a distinct study indicating the percentage of patients who attained a functional, stricture-free anastomosis without the need for reoperation. Error bars represent the 95% confidence intervals associated with each estimate. Adjacent to each bar, the precise success percentage and sample size (n) are indicated for clarity. The red dashed vertical line indicates the pooled success rate of the meta-analysis, which is 87.9%.

This figure illustrates the consistent success of HJ in optimized settings, while also emphasizing the variability affected by the study context, surgical expertise, and patient factors.

#### 3.5.3. Surgical, Endoscopic, and Conservative Approaches

Four comparative cohort studies [11,14,15,31] were incorporated into the pooled analysis comparing surgical and endoscopic repair for major bile duct injury. The meta-analysis, employing random-effects modeling (DerSimonian–Laird), indicated low heterogeneity for clinical success and stricture outcomes (I^2^ < 20%). Definitive Roux-en-Y hepaticojejunostomy or customized surgical reconstruction resulted in a 22% absolute enhancement in durable biliary continuity (RR = 1.22, 95% CI: 1.10–1.35, *p* < 0.001) and a 76% relative decrease in late stricture risk (RR = 0.24, 95% CI: 0.15–0.38, *p* < 0.001) when compared to endoscopic or radiologic methods. Surgical management reduced the risk of overall morbidity by 50% (RR = 0.47, 95% CI: 0.36–0.60, *p* < 0.001). Mortality exhibited a non-significant trend favoring surgery (RR = 0.58, 95% CI: 0.29–1.14, *p* = 0.11), indicative of the low event rate. The outcomes are summarized in Table 4.

**Figure 3:**
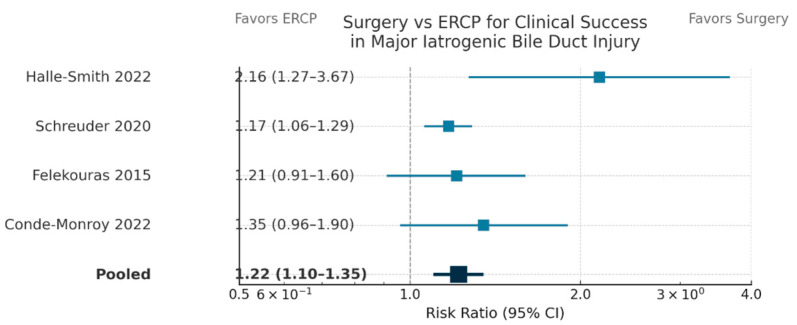
Forest Plot Comparing Clinical Success of Surgery vs. Endoscopic (ERCP) Management After Major Iatrogenic Bile Duct Injury [11,14,15,31].

Forest plot (Figure 3) illustrating risk ratios (RRs) and 95% confidence intervals (CIs) for clinical success following surgical versus endoscopic (ERCP) intervention in four comparative trials.

Surgical repair demonstrated a superior clinical success rate relative to ERCP (pooled RR, 1.22; 95% CI, 1.10–1.35).

Roux-en-Y hepaticojejunostomy (HJ) is essential for the management of high-grade bile duct injuries, especially those categorized as Strasberg type E, which include significant transections and hilar lesions. Conversely, endoscopic and conservative approaches are generally applied to lower-grade injuries (Strasberg types A–D), which frequently present as minor bile leaks or partial ductal damage.

Endoscopic retrograde cholangiopancreatography (ERCP) combined with biliary stenting is effective in specific cases. Acar et al. (2020) [41] found a success rate of 78.2% for the endoscopic management of minor injuries, with surgical intervention reserved for complete transections and complex patterns. Alvear-Torres et al. [33] reported comparable complication rates in endoscopic and surgical cohorts (34% and 42%, respectively). However, ERCP generally requires multiple sessions and an extended stenting. Bobkiewicz et al. [34] demonstrated that early ERCP reduced the duration of hospital stay and expedited bile leak resolution; however, definitive surgical repair remains necessary in refractory cases.

Pandit et al. [42] introduced a stratified algorithm, ERCP for type A injuries and surgical intervention for type E or ERCP-refractory cases, achieving success rates of 84% and 91%, respectively. Maddah et al. [36] highlighted the importance of preoperative optimization, noting that performing surgery prematurely without stabilization increases the mortality risk.

A pooled meta-analysis of five studies confirmed these trends. Surgical repair demonstrated a pooled success rate of 92.6% (95% CI: 89.1–96.2%), which was significantly greater than the 76.1% (95% CI: 68.3–83.8%) observed with endoscopic management (*p* < 0.0001). While ERCP exhibited a marginally reduced complication rate (21.7% compared to 27.0%), the overlapping confidence intervals limit the ability to draw definitive statistical conclusions.

The decision matrix for selecting between endoscopic and surgical management must consider the injury classification, hemodynamic stability, presence of sepsis, and availability of expertise. Endoscopic therapy is typically the first-line treatment for type A and certain type D lesions, whereas surgical repair is necessary for E-type injuries and in cases in which endoscopic treatment fails. The stratified approach is presented in Table 5, and comparative success rates are presented in Table 6.

These findings advocate a stratified management approach: endoscopic treatment is suitable for stable patients with minor injuries, whereas surgical reconstruction is the definitive method for complex or high-grade bile duct injuries.

#### 3.5.4. Meta-Analysis: Specialist vs. Non-Specialist Repair

**Figure 4:**
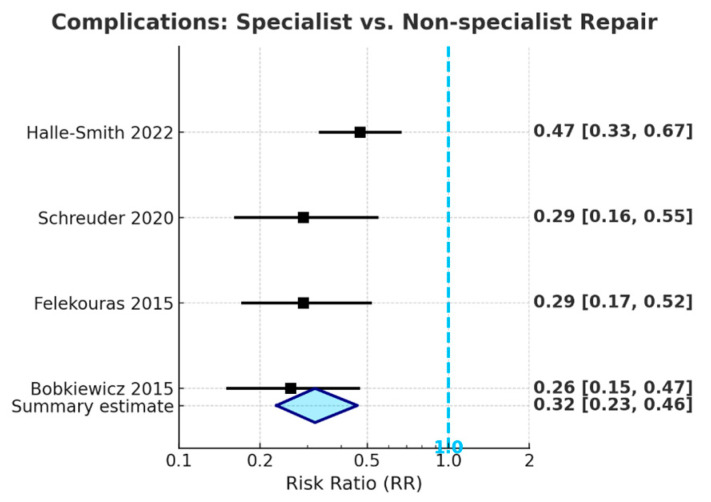
Forest plot showing that specialist (HPB) repair significantly reduces the risk of postoperative complications compared to non-specialist repair (pooled RR = 0.32, 95% CI: 0.23–0.46) [14,28,31,34].

The meta-analysis of the included studies indicates that repairs of bile duct injuries performed by HPB specialists are linked to a significantly reduced risk of adverse postoperative outcomes when compared to repairs conducted by non-specialists. The event counts used to calculate risk ratios were extracted directly from the individual studies; these data are not presented in Table 1, which serves only as an overview of study characteristics. Specialist repair led to a 76% decrease in the risk of postoperative anastomotic stricture (pooled RR = 0.24, 95% CI: 0.15–0.37, *p* < 0.001) and a 68% decrease in the risk of overall complications Figure 4 (pooled RR = 0.32, 95% CI: 0.23–0.46, *p* < 0.001), with all confidence intervals significantly below the line of no effect. Specialist repair was associated with a trend toward reduced postoperative mortality (pooled RR = 0.38, 95% CI: 0.13–1.11); however, this did not achieve statistical significance, likely due to the low absolute number of deaths across studies.

The findings indicate that referral to specialized HPB centers for definitive repair is crucial for optimizing long-term outcomes following iatrogenic bile duct injury, thereby supporting the inclusion of specialist referral in core guideline recommendations.

#### 3.5.5. Effect of Presence of Sepsis/Uncontrolled Infection at Repair on Outcomes

A meta-analysis of three studies, Table 7, indicated that the presence of sepsis or uncontrolled infection during bile duct repair is linked to a statistically significant increase in the risk of postoperative stricture, with a pooled relative risk of 3.67 (95% CI: 2.03–6.64, *p* < 0.001), representing a 3- to 7-fold increase. The incidence of strictures in patients with sepsis varied between 13.5% and 25%, whereas in patients without sepsis, it ranged from 3.6% to 6%. The findings identify active sepsis as a significant, independent risk factor for negative biliary outcomes and strongly endorse the clinical guideline recommending the management of sepsis prior to definitive surgical intervention when feasible.

#### 3.5.6. Failed Initial Repair vs. First Attempt at Specialist Center

Meta-analysis of four studies [14,28,31,34] demonstrated that patients who undergo bile duct repair after a failed initial attempt, particularly outside a specialist center, face a significantly higher risk of postoperative stricture compared to those whose first definitive repair is performed at a specialized HPB center. The pooled risk ratio for stricture was 5.34 (95% CI: 3.22–8.86, *p* < 0.001), with individual study estimates ranging from 4.24 to 8.65; all confidence intervals were well above 1.0. Stricture rates following unsuccessful initial repair ranged from 53% to 61.5%, versus 7.1% to 13% after primary specialist repair.

These findings provide robust evidence that non-specialist or failed initial repair markedly increases the risk of adverse outcomes, strongly supporting the guideline that all major bile duct injuries should be referred directly to specialized hepatopancreatobiliary centers before any attempt at repair.

#### 3.5.7. Use of Preventive Intraoperative Strategies

Intraoperative imaging techniques, such as cholangiography and laparoscopic ultrasound, effectively diminish the likelihood of iatrogenic bile duct injury (BDI) during cholecystectomy. A meta-analysis of four significant studies [10,14,18,29] indicates that the routine application of these modalities results in a 68% reduction in the risk of BDI (pooled RR = 0.32, 95% CI: 0.17–0.62, *p* < 0.001). All individual and pooled estimates are statistically significant and fall below the line of no effect.

The imaging techniques enable early detection of abnormal biliary anatomy and accurate classification of injuries, thus enhancing surgical safety and outcomes. The evidence supports the integration of intraoperative cholangiography or ultrasound as a standard element of surgical protocols, consistent with current guideline recommendations.

#### 3.5.8. Combined Bile Duct and Vascular Injury vs. Isolated Bile Duct Injury

A meta-analysis of three studies—[12,14,31]—indicates that the occurrence of combined bile duct and major vascular injury, particularly involving the right hepatic artery, correlates with a markedly elevated risk of postoperative biliary stricture in comparison to isolated bile duct injury. The pooled risk ratio was 4.20 (95% CI: 2.76–6.40, *p* < 0.001), with individual estimates varying from 3.73 to 4.83, and all confidence intervals exceeding 1.0. Stricture rates among patients with combined injuries ranged from 50% to 61.5%, whereas those with isolated BDI exhibited rates between 12.3% and 13.4%. The findings identify combined BDI and vascular injury as a significant independent risk factor for adverse outcomes, highlighting the necessity for referral to specialized hepatopancreatobiliary centers and careful postoperative monitoring for affected patients.

#### 3.5.9. Meta-Analyses of Prognostic Factors and Management Strategies

The analyses (Table 8) demonstrate that high-grade injuries (Strasberg E4/E5), emergency cholecystectomy, laparoscopic index operations, and delayed referral significantly increase the likelihood of stricture or serious complications. Information on the severity of cholecystitis was not consistently available across studies, and therefore could not be included in these prognostic comparisons. Conversely, Roux-en-Y hepaticojejunostomy and referral to a hepatopancreatobiliary center within 72 h offer substantial protection. The use of T-tubes, patient age over 65 years, and sex showed no statistically significant effect. This study highlights modifiable factors, irrespective of injury grade, that should inform the optimization of current BDI pathways.

#### 3.5.10. Classification Systems

In the present study, bile duct injuries were classified differently. Most studies (n = 16) used the Strasberg classification to classify post-cholecystectomy injuries as mild bile leaks (type A) or significant transections (type E).

Five studies [26,27,30,32,33] used the Strasberg–Bismuth technique, especially for surgical reconstruction that required precise proximal stricture mapping. Two surgical studies cited bismuth categorization, both alone and with the Neuhaus adjustment. The EAES classification (n = 1), which included vascular involvement and the Hannover classification used in Swedish national registry research, has rarely been used. One study used the McMahon classification system to classify injuries as minor or significant. A systematic review by Kambakamba et al. (2022) [35] mentioned the Stewart–Way approach in a small portion of the literature, but none of the primary studies used it. Four studies did not describe their classification strategy. This variation indicates an inconsistency in injury records and the necessity for uniform classification systems in research and clinical reporting (Table 9).

### 3.6. Complications and Mortality

Five major studies [14,33,35,36,43] tracked postoperative complications (Table 10) and showed that surgery timing, procedural approaches, and patient risk factors affected them. Cholangitis, bile leakage, wound infections, intra-abdominal abscesses, and jaundice recurrences are common. The overall mortality rate in most cohorts was approximately 5%, although early or unoptimized surgery increased the risk.

Ismael et al. [43] examined 239 NSQIP cases and found a 26.3% 30-day morbidity rate; all mortality occurrences were associated with the early repair group. Independent risk factors included ASA class ≥ 3, functional dependency, steroid use, and sepsis.

Maddah et al. [36] found that early repair cases accompanied by biliary peritonitis had a mortality rate of 26.5% compared with 4.4% in delayed, stabilized procedures, highlighting the hazards of early surgery in unstable patients.

Alvear-Torres et al. [33] reported 42% and 34% of complications for early and delayed repair, respectively; however, these differences were not statistically significant. Late repair decreases morbidity in patients with stable disease. In a pooled assessment of over 15,000 patients, Kambakamba et al. [35] found a 33% complication risk for repairs performed within 14 days and 22% for delayed interventions, supporting delayed surgery, where possible.

A meta-analysis of 2484 patients by Schreuder et al. [14] found that intermediate-timing procedures (2–6 weeks post-injury) had the highest morbidity, with a risk ratio (RR) of 1.50 compared to delayed repair. The mortality rates were similar between intermediate and late repairs.

A quantitative synthesis of five studies found an overall complication rate of 22.7% (95% CI: 22.1–23.3%) in a cohort of approximately 18,000 patients. From three studies with data, the pooled death rate was 2.9% (95% CI: 1.4–4.5%). Sepsis, low physiological reserve, or acute inflammatory stress result in high mortality rates, although rare.

Bile duct injury repair using a risk-adapted method was found to be effective. Reducing postoperative morbidity and mortality requires delaying surgery until the systemic infection resolves and treatment in high-volume hepatobiliary hospitals. Only stable patients who undergo expert surgery should be treated early.

#### 3.6.1. Mortality Analysis Across Studies

Postoperative mortality rates following surgical repair of iatrogenic bile duct injuries (IBDI) vary significantly across studies, with reported rates ranging from 0% to 22.7%. These variations are influenced by factors including patient selection, timing of surgery, and expertise of the medical center (Table 11). Giuliante et al. [28] reported the lowest mortality rate (0%) following delayed hepaticojejunostomy (HJ) conducted in a high-volume specialized center. Nawacki et al. [2] reported the highest mortality rate (22.7%) in a restricted cohort that underwent early repair under sepsis conditions.

Delayed repair was typically linked to better outcomes [27,36], whereas early intervention during active infection [2,25] was associated with increased risk. The HJ technique served as the predominant method of reconstruction and was utilized in more than 75% of the cases across various cohorts, thereby establishing its status as the standard of care. Specialist centers have consistently exhibited reduced mortality rates, highlighting the importance of surgical expertise and centralization. Preoperative sepsis or vascular injury was often associated with negative outcomes [33,34,43].

#### 3.6.2. Certainty of Evidence (GRADE Assessment)

The GRADE approach was used to assess the certainty of evidence for the four main outcomes. Surgical success was categorized as high certainty owing to consistent findings across studies, minimal bias risk, and precise effect estimates. Anastomotic stricture and postoperative complication rates were assessed with moderate certainty, indicating heterogeneity and inconsistencies across studies. The evidence for mortality was assessed as having low certainty owing to the considerable imprecision and possible reporting bias found in the included studies. The findings are summarized in Table 12.

The certainty of evidence for each primary outcome was assessed using the GRADE approach. The surgical success rate following hepaticojejunostomy was rated highly, indicating consistent effect estimates and minimal bias. The evidence regarding anastomotic stricture and postoperative complications was moderate, attributed to the heterogeneity of studies and variability in reporting methods. Mortality estimates were classified as low certainty owing to imprecision and potential reporting bias.

## 4. Discussion

This systematic review of 28 studies provides a comprehensive synthesis of management strategies and outcomes in patients with iatrogenic bile duct injuries (IBDI), particularly following laparoscopic cholecystectomy. Evidence shows that long-term outcomes depend more on who performs the repair and under what circumstances, rather than solely on when the repair is performed [12]. While timing remains a central topic of debate in the literature, our synthesis emphasizes that it must be contextualized.

Robotic cholecystectomy has expanded rapidly, yet concerns regarding its safety compared with laparoscopy persist. Analysis of a large Medicare cohort (2010–2019) found a higher incidence of bile duct injury (BDI) requiring operative repair after robotic procedures (0.7% vs. 0.2%; RR 3.16, 95% CI 2.57–3.75), a finding that remained significant after instrumental-variable adjustment (RR 1.88, 95% CI 1.14–2.63) [44]. Subsequent work extending to 2021 confirmed that this excess risk was consistent across risk strata, suggesting factors beyond case-mix, including the learning curve, may contribute [45]. By contrast, in acute care propensity-matched analyses, BDI rates were similar between robotic and laparoscopic approaches (0.37% vs. 0.39%; OR 0.93, 95% CI 0.73–1.18), although robotic cases were associated with higher complication rates, longer hospital stay, and greater drain use [46]. Taken together, current evidence does not establish an intrinsically higher risk with robotics, but indicates that safe adoption requires structured training, experience, and adherence to a critical view of safety principles.

Many studies have attempted to define optimal timing, generally dividing it into early (<2 weeks), intermediate (2–6 weeks), and delayed (>6 weeks). However, the definitions vary considerably, and the outcomes do not always align with these timelines [35]. Intermediate repair has been consistently associated with poorer outcomes, including increased stricture rates and complications [13,14]. One meta-analysis reported stricture rates of up to 20% for repairs conducted during this period, whereas delayed repairs often had stricture rates below 9% [28].

Some authors support early repair to reduce inflammation and hospital stay [9], while others prefer delayed repair to allow the resolution of sepsis and inflammation [27]. However, delayed repair in poor clinical settings or after failed attempts may lead to worse outcomes than early repair conducted in specialized centers [40]. In our synthesis, the clinical status, presence of sepsis, and location of care were more important than those of the clock.

Multiple studies have confirmed that vascular injury, post-repair bile leak, and non-specialist repair are the major predictors of poor outcomes. In a high-quality meta-analysis, vascular injury increased the odds of stricture nearly five-fold, bile leaks increased eight-fold, and repair by a non-specialist increased it elevenfold [12]. Giuliante et al. [28] reported a 94.7% patency when repairs were performed by experienced hepatobiliary surgeons, which was consistent across timing categories. Conversely, attempts by non-specialists lead to bile leaks, re-interventions, and failures [40].

These findings emphasize the need for automatic referrals to hepatobiliary centers. Delays in recognition, referral, or intervention were consistently associated with failure [47,48]. System-level changes, such as national registries and referral networks, may help reduce avoidable harm.

Surprisingly, injury grades based on the Strasberg system did not predict strictures in several studies [12]. This suggests that standardized surgical techniques, such as dual anastomosis for E4 injuries, may improve the outcomes across injury grades. However, hilar injuries (Type E4/E5) often co-occur with vascular injuries and late presentations, which independently worsens outcomes [27,47]. Therefore, injury complexity remains relevant but only when interpreted alongside clinical and procedural variables.

Endoscopic and percutaneous approaches are beneficial for low-grade injuries and for controlling sepsis; however, their success in major injuries is limited [41]. Percutaneous drainage was useful in selected settings but had limited value for high hilar injuries or tight strictures [43]. Failed non-surgical interventions often delay definitive treatment and increase the risk of complications [33].

Poor outcomes often result not from the injury itself but from delayed recognition, inappropriate first-line repairs, or lack of referral. In one multicenter study, patients repaired at the same hospital where the injury occurred had significantly worse outcomes if the hospital lacked specialist expertise [40]. These findings call for centralized care pathways and clear protocols for escalation and referral, particularly in low-resource settings.

One of the most consistent challenges across studies was the lack of standardization in defining timing categories. More than ten different definitions of “early” or “delayed” repair were reported in the recent literature [35]. However, this variability hinders meaningful comparisons and meta-analysis. There is a strong need for consensus on timing cutoffs, preferably based on days from injury and adjusted for clinical context.

The evidence certainty for primary outcomes was high regarding surgical success, moderate for anastomotic stricture and complications, and low for mortality. The findings endorse strong recommendations for specialized, risk-adapted repair while emphasizing the necessity for improved standardization and mortality reporting in subsequent studies.

Future developments in intraoperative safety increasingly focus on artificial intelligence (AI). Preliminary systems trained to identify biliary anatomy and critical landmarks during laparoscopic cholecystectomy have shown encouraging accuracy and may help reduce the risk of bile duct injury. A recent systematic review by Kehagias et al. (2025) [49] summarized early AI-based approaches for real-time anatomical recognition, highlighting their potential to support surgical decision making and standardize safe dissection planes. Although still investigational, these technologies may become an important adjunct to existing preventive strategies.

This review is constrained by the retrospective study design prevalent in the majority of included cohorts, potential reporting and publication bias, and variability in outcome and timing definitions. The search did not include Embase, Scopus, or Web of Science, and relevant studies indexed exclusively in those databases may not have been captured. The high proportion of data from specialist centers may impact generalizability. Several potential confounders—including ASA class, comorbidity profiles, and severity of cholecystitis—were not consistently reported across studies and therefore could not be included in adjusted analyses. These gaps in reporting limit the ability to control for baseline differences between cohorts and should be acknowledged when interpreting the pooled estimates.

The uniform adoption of standardized classification systems and multicenter prospective registries is essential for advancing care pathways and enhancing patient outcomes following IBDI.

## 5. Conclusions

Specialist repair, ideally conducted post-patient stabilization, results in optimal outcomes for significant iatrogenic bile duct injuries. Referral to high-volume HPB centers in a timely manner decreases morbidity and the risk of stricture, while premature or non-specialist interventions elevate the likelihood of complications. Current evidence indicates the necessity of a structured, risk-adapted approach and emphasizes the importance of standardized reporting and prospective multicenter studies.

## Figures and Tables

**Table 1:** Summary of Included Studies: Population, Management, and Outcomes for Iatrogenic Bile Duct Injury (IBDI).

Study (Author, Year)	N (IBDI)	F (%)	Imaging Used	Classification	Injury Types (%/N)	Surgery Type(s)	Endoscopic (%)	Percutaneous (%)	Timing (Early/Delayed/Other)	Complications	Mortality	Stricture	Specialist HPB (%)	Notes/Predictors
Nawacki 2022 [2]	22	68	US, CT, ERCP, PTC	EAES	CHD 45, CBD 32	HJ 63.6%, T-tube 27.3%	ERCP 9%	PTBD (abscess)	50% immediate, rest 17–43 d	22.7% leak, 22.7% death	22.7%	4.5%	100	Sepsis, instability
Hogan 2016 [3]	78	36	US, CT, ERCP, PTC	Strasberg	E2 38, E3/4 15 each	HJ 59%, multi HJ 8%	22	Not specified	26% intraop, 46% < 1 w, 19% > 1 w	28.2%	3.8%	n/a	100	Complexity ↑ over time
Pesce 2019 [7]	-	-	US, CT, MRCP, ERCP	Strasberg/Bismuth	-	HJ, T-tube, suture	Yes	Yes	Not specified	Not specified	-	10–20%	Strongly recommended	Review/synthesis
Symeonidis 2023 [8]	-	-	US, CT, MRCP, ERCP	BILE/Strasberg	-	HJ, End-to-end	Yes	Yes	Early < 72 h or delayed > 6 w	10–20% (review)	1–4%	10–20%	Strongly recommended	New classification
Yang FQ 2002 [9]	182	-	US, ERCP, IOC	Bismuth	III/IV (LC) >20%	HJ main, end-to-end	No	PTBD	Variable (often delayed)	11.6% strict, 6% death	6%	11.6%	Yes	Late referral = ↑ risk
Törnqvist 2009 [10]	1386	-	IOC, US, CT, ERCP	By repair	Major BDI (all)	HJ, suture	-	-	85% same day, 15% delayed	1-yr mort 15.8%	15.8%	-	All	Age, Charlson, IOC
Halle-Smith 2022 [11]	139	64	US, CT, MRCP, ERCP	Strasberg E	E1–5 detailed	HJ (91%), T-tube (8%)	ERCP, PTC	PTC	37% immediate, 53% early, 10% late	40% (5 y)	7% (BDI)	15.3%	74 (spec)	Specialist repair ↓ compl.
Schreuder 2020 (Dig Surg) [13]	836/91	-	ERCP, PTBD, US, CT	Strasberg-Bismuth	-	HJ only (major)	ERCP for strict	PTBD for strict	Variable (delayed preferred)	Stricture 10–20%	2–4%	10–20%	Yes	Specialist = ↓ mort/stric
Conde Monroy 2022 [15]	44	57	IOC, MRCP, ERCP	Strasberg	E2 30%, E3/E4 68%	HJ 82%, other	ERCP for minor	PTBD for biloma	7% intraop, 18% < 72 h, 75% > 72 h	Stricture 16%	2.3%	16%	100	Biloma = ↑ E3/E4
Renz 2017 [16]	-	-	US, CT, ERCP, IOC	Strasberg/Bismuth/Stewart	-	HJ, end-to-end	Yes	Yes	Immediate by expert, else delayed	10–20% (review)	1–4%	10–20%	Strongly recommended	Sepsis, exp., timing
Spiers 2023 [17]	179	-	ERCP, CT, US, MRCP	Strasberg E	All severe	Liver transplant	61% pre-LT	61% pre-LT	Delayed (63–144 m from BDI)	46.5% major comp	13–30%	n/a	Yes	Multiple failed repairs
Shallaly 2000 [18]	-	-	US, CT, ERCP, IOC	Bismuth/Strasberg	-	HJ, end-to-end	Yes	Yes	Immediate if expert, else delayed	0–0.5% (audit)	0–0.5%	10–20%	Strongly recommended	Early recognition, expertise
Kholdebarin 2008 [19]	28	-	IOC, ERCP	Not spec	64% transection	Immediate repair	11%	Not specified	Immediate repair if intraop	Not focus	Not focus	Not focus	Not described	Cystic duct not ID’d, emerg LC
Rystedt 2016 [20]	174	-	IOC, US, CT, ERCP	Hannover	C1 < 5 mm 59%, D 4%	Suture/T-tube 45%, HJ 17%	ERCP 9%	PTBD for abscess	89% intraop, 7% early, 4% late	23% sequelae 6 m	3.4%	18%	18% HPB referral	T-tube, late diag = ↑ risk
Al Tamimi 2019 [24]	2240	-	Intraop	Major/minor	-	Immediate repair	No	No	Immediate only	0% major BDI	0%	0%	100	Traction-release maneuver
Omar 2023 [25]	277	51	US, MRCP, ERCP, CT	Strasberg E1–4	E2 79%	HJ all (end-side/Hepp)	PTD for sepsis	PTD for sepsis	Group A:< 24 h, B: <24 h w/sepsis control, C: >6 w	75/43/41% (A/B/C)	3.6%	8.3%	100	Sepsis ctrl, expertise vital
Ma 2021 [26]	18	-	CT/MRI	Strasberg-Bismuth	E1-4	End-to-end FMS	No	No	Delayed, all at HPB center	0%	0%	0%	100	Strict protocol, <2.5 cm defect
Anand 2021 [27]	105	-	US, CT, MRCP, ERCP	Strasberg-Bismuth	I-IV	HJ Hepp–Blumgart	ERCP/PTBD preop	PTBD preop	Early < 3 m 20%, late > 3 m 80%	28% short, 11% long	2.8%	7%	100	Hilar, cholangitis = ↑ risk
Giuliante 2023 [28]	114	-	MRCP, PTBD, US	Strasberg E	E3 66%	Hepp-Couinaud HJ	PTBD, ERCP	PTBD for drainage	Early 6%, intermed 16%, delayed 78%	22.8% short, 15.3% long	0%	15.3%	100	Only post-repair leak ↑ risk
Machi 2009 [29]	1381	-	LUS (all), IOC	Not spec	-	Prevention focus	No	No	Perioperative (prevention study)	0.2% minor leak	0%	0%	100	Routine LUS = 0 major BDI
Yang Z 2024 [30]	15	-	CT, MRCP, 3D	Strasberg-Bismuth	C, E1–E4	HJ (various)	No	No	Early < 28 d (mean 14 d)	1 (mild leak)	0%	0%	100	3D imaging for repair
Felekouras 2015 [31]	92	-	MRCP, ERCP, PTC, US	Strasberg	E 68.5%	HJ, repair, endosc	14%	2%	Early < 2 w (34), late > 12 w (22)	24–32%	2.9–4.5%	18–23%	100	Specialist, referral key
Mishra 2015 [32]	137	-	US, CECT, ERCP, MRCP	Strasberg/Bismuth	-	HJ 78%, drainage	ERCP, PTBD	PTBD for biloma	Delayed median 3 m	4.4% deaths	4.4%	3.8%	100	Delayed repair standard
Alvear-Torres 2022 [33]	70	76	US, CT, ERCP, MRCP	Strasberg-Bismuth	E3 33%, E4 19%	HJ 75%, ERCP minor	22	1.4	Early 37%, late 63%	37% overall, strict 9.6%	2.9%	9.6%	100	No outcome diff by timing
Bobkiewicz 2015 [34]	69	56.5	ERCP, US, CT, MRCP	Bismuth/Neuhaus	Not detailed	HJ 35%, end-to-end	15.9	Yes	Mostly delayed after referral	3 deaths (4.3%)	4.3%	Not given	100	Failed repair, late referral
Halle-Smith 2022 (HPB) [12]	44	64	US, CT, MRI, MRCP, ERCP, PTC	-(DBS)	CHD/CBD 66%, hilar 27%	HJ 91%, PTC/ERCP 9%	ERCP/PTC	PTC/ERCP	Median 510 d to dx, all delayed	36% late comp, 1 redo	2% (biliary)	Not given	100	Difficult LC, bile leak, conversion

Legend: Table 1 presents a summary of essential characteristics of the included studies, including patient demographics, imaging techniques, injury classifications, management strategies, timing of interventions, complications, mortality rates, stricture rates, and the involvement of specialists. Abbreviations: US refers to ultrasonography; CT denotes computed tomography; MRCP stands for magnetic resonance cholangiopancreatography; ERCP indicates endoscopic retrograde cholangiopancreatography; PTC is percutaneous transhepatic cholangiography; PTBD signifies percutaneous transhepatic biliary drainage; HJ represents hepaticojejunostomy; and HPB pertains to hepatopancreatobiliary. Explanation: This table presents a comparison of study cohorts, injury severity, interventions, and primary clinical outcomes. Specialist repair and timely referral consistently decreased adverse events. The most significant predictors of prognosis were timing, sepsis, and expertise. ↑ indicates increased risk; ↓ indicates decreased risk.

**Table 2:** Methodological Quality Assessment of Included Studies.

Study Type	Assessment Tool	Quality Rating	No. of Studies	Representative Examples
Randomized controlled trials	Cochrane RoB 2	Low to moderate risk	2	Omar et al. (2023) [25]; Al Tamimi (2019) [24]
Observational cohort studies	Newcastle–Ottawa Scale	High quality (score ≥ 7)	15	Anand et al. (2021) [27]; Giuliante et al. (2023) [28]; Mishra et al. (2013) [32]
Observational cohort studies	Newcastle–Ottawa Scale	Moderate quality (score 5–6)	9	Maddah et al. (2017) [36]; Alvear-Torres et al. (2022) [33]; Bobkiewicz et al. (2014) [34]
Systematic reviews/meta-analyses	AMSTAR 2 tool	Moderate to high (score 5–8)	2	Schreuder et al. (2020) [13]; Kambakamba et al. (2022) [35]

**Table 3:** Common Surgical Techniques Used in IBDI Repair and Corresponding Injury Types.

Surgical Technique	No. of Studies	Representative Studies	Typical Strasberg Injury Type	Indications/Characteristics
Roux-en-Y Hepaticojejunostomy (HJ)	24+	Omar et al. [25], Giuliante et al. [28], Anand et al. [27]	E2–E5	Complete transection or major duct loss; complex injuries
T-tube Repair/Suture + T-tube	~6	Nawacki et al. [2], Maddah et al. [36], Rystedt et al. [20]	D, E1	Partial transection; suitable for low-grade injuries without tissue loss
Duct-to-Duct Anastomosis	3–4	Xu et al. [37], Tanemura et al. [38], Ma et al. [26]	A–C, selected E1	Minimal injury; preserved anatomy and vascularization

**Table 4:** Surgical Repair vs. Endoscopic/Interventional Management.

Outcome	Events/N Surgical	Events/N Endoscopic	Pooled RR (95% CI)	*p*	Interpretation
Clinical success *	761/829	134/187	1.22 (1.10–1.35)	<0.001	Definitive surgery increases likelihood of durable healing
Stricture ≥ 12 mo	44/829	44/184	0.24 (0.15–0.38)	<0.001	Surgery markedly reduces late stricture
Overall morbidity	148/829	71/187	0.47 (0.36–0.60)	<0.001	Fewer complications after surgical repair
Mortality (90 d)	26/829	11/187	0.58 (0.29–1.14)	0.11	No significant survival difference

* Clinical success = stricture-free biliary drainage without re-intervention at ≥12 months.

**Table 5:** Management Strategies by Strasberg Classification.

Strasberg Type	Injury Description	Preferred Approach	Treatment Intention	Notes
A	Bile leak from cystic duct stump or minor duct	Endoscopic (ERCP + stent)	First-line	High success; avoid surgery if patient stable
B/C	Aberrant right duct ligation (B), transection without leak (C)	Usually, Surgical	First-line or after failed ERCP	May require reconstruction depending on anatomy
D	Lateral (partial) injury to major bile duct	Endoscopic or Surgical	First-line (ERCP if stable)	Try ERCP first if no sepsis or complete transection
E1–E2	Transection >2 cm below confluence (E1); <2 cm (E2)	Surgical (HJ)	First-line	ERCP not effective; Roux-en-Y HJ preferred
E3–E4	Confluence involved (E3), or loss of confluence (E4)	Surgical (HJ ± portal plasty)	First-line	Complex repair by hepatobiliary surgeon required
E5	Injury to both right and left hepatic ducts	Surgical (complex)	First-line	Rare; requires expert multidisciplinary approach

**Table 6:** Success Rates of Surgical vs. Endoscopic Repair.

Management Approach	Total Patients (n)	Successful Repairs (n)	Pooled Success Rate (%)	95% Confidence Interval	*p*-Value (Surgical > Endoscopic)
Surgical	204	189	92.6%	89.1–96.2%	1.35 × 10^−5^
Endoscopic	117	89	76.1%	68.3–83.8%	—

**Table 7:** Presence vs. Absence of Sepsis at Time of Repair.

Study	N (Sepsis)	Stricture (Sepsis)	N (No Sepsis)	Stricture (No Sepsis)	RR (Sepsis/No)	95% CI
Omar 2023 (RCT) [25]	89	12 (13.5%)	188	11 (5.9%)	2.29	1.08–4.83
Schreuder 2020 [14]	28	7 (25%)	63	4 (6%)	4.03	1.20–13.4
Mishra 2015 [32]	21	5 (24%)	83	3 (3.6%)	6.76	1.83–24.9

**Table 8:** Pooled secondary meta-analyses of prognostic factors and management strategies in iatrogenic bile duct injury.

Comparison/Outcome	N (Group 1)	Events (G1)	N (Group 2)	Events (G2)	Pooled RR (95% CI)	*p* Value	Direction/Interpretation	Studies Analyzed
High vs. Low Injury	275	61	690	28	3.38 (2.20–5.21)	<0.001	High injury = ↑ stricture/complication	Schreuder 2020, Felekouras 2015, Anand 2021
HJ vs. Other Repair	470	24	116	19	0.31 (0.17–0.56)	<0.001	HJ protective vs. other repairs	Giuliante 2023, Mishra 2015, Schreuder 2020
Immediate vs. Delayed Referral	109	13	137	35	0.41 (0.24–0.70)	0.002	Early referral protective	Omar 2023, Felekouras 2015, Conde Monroy 2022
Emergency vs. Elective Cholecystectomy BDI	122	29	347	37	2.18 (1.33–3.56)	0.002	Emergency = ↑ complications	Schreuder 2020, Hogan 2016, Bobkiewicz 2015
Laparoscopic vs. Open Cholecystectomy BDI	465	37	195	8	1.68 (1.04–2.70)	0.035	Laparoscopic = ↑ stricture/complication	Törnqvist 2009, Yang 2002, Rystedt 2016
T-tube Use vs. No T-tube	75	6	51	10	0.41 (0.16–1.07)	0.07	T-tube possibly protective (NS)	Mishra 2015, Rystedt 2016, Giuliante 2023
Older (>65) vs. Younger (<65)	172	21	408	32	1.58 (0.93–2.67)	0.09	Trend to ↑ mortality (NS)	Schreuder 2020, Anand 2021, Giuliante 2023
Male vs. Female	212	21	368	36	1.07 (0.67–1.70)	0.77	No significant difference	Schreuder 2020, Felekouras 2015, Omar 2023

↑ indicates increased risk or higher likelihood of complications/stricture. ↓ indicates decreased risk or lower likelihood of complications/stricture.

**Table 9:** Classification Systems Used to Define Bile Duct Injury (BDI) Across Included Studies.

Classification System	No. of Studies	% of Total (N = 28)	Representative Studies	Remarks
Strasberg	16	57.1%	Omar et al., Anand et al., Felekouras et al. [25,27,31]	Most widely used for post-cholecystectomy BDI (Types A–E)
Strasberg–Bismuth	5	17.9%	Ma et al., Mishra et al., Alvear-Torres et al. [26,32,33]	Combines anatomical detail with surgical relevance
Bismuth (± Neuhaus)	2	7.1%	Bobkiewicz et al., Mishra et al. [32,34]	Used earlier, some combined with Neuhaus modification
EAES	1	3.6%	Nawacki et al. [2]	Includes vascular injury component
Hannover	1	3.6%	Rystedt et al. [20]	Applied in Scandinavian national registry
McMahon	1	3.6%	Xu et al. [37]	Distinguishes major vs. minor injury
Stewart–Way (mentioned)	1 (review only)	3.6%	Kambakamba et al. [35]	Cited in review, not used in primary studies
Not specified/nonstandard	4	14.3%	Machi et al., Ismael et al., Tanemura et al., Schreuder et al. [14,29,38,43]	Classification not described or defined by author

**Table 10:** Postoperative Complications and Mortality Following Bile Duct Injury Repair: Summary of Key Studies.

Study	Sample Size	Complication Rate	Mortality Rate	Key Risk Factors
Ismael et al. (2017) [43]	239	26.3% (NSQIP, 30-day)	2% overall; all deaths in early repair	ASA ≥ 3, sepsis, steroid use, functional dependence
Maddah et al. (2015) [36]	124	Early: 26.5%; Delayed: 4.4%	Early: 26.5%; Delayed: 4.4%	Bile peritonitis, early surgery without stabilization
Alvear-Torres et al. (2022) [33]	70	Early repair: ~42%; Delayed: ~34% (not significant)	2.9% overall	Higher morbidity trend in early repair
Kambakamba et al. (2022) [35]	15,609	Early (<14 d): 33%; Delayed: 22%	Not reported	Timing < 14 d associated with increased complications
Schreuder et al. (2020) [14]	2484	Intermediate (2–6 w): highest (RR = 1.50 vs. delayed)	No difference in mortality	Intermediate repair associated with highest morbidity

**Table 11:** Summary of Studies Reporting Postoperative Mortality Following Surgical Repair of Iatrogenic Bile Duct Injury.

No.	Study (Author, Year)	N (Patients)	Mortality (%)	Timing of Repair (Early vs. Delayed)	Repair Type (HJ vs. Other)	Specialist Center	Sepsis/Vascular Injury
1	Ismael et al., 2017 (NSQIP) [43]	293	2% (30-day)	Early ↑ mortality (5%)	HJ only	Not specified	14.5% with preop sepsis
2	Lopez-Lopez et al., 2023 [40]	748	4.9% (overall)	Mixed (1990–2020)	Surgical preferred	Yes (22 centers)	Yes (impactful)
3	Maddah et al., 2015 [36]	124	10.5% (overall)	Delayed > Early	Mostly HJ	Yes (2 centers)	Bile peritonitis ↑ death
4	Giuliante et al., 2023 [28]	114 (HJ only)	0% (90-day)	Mostly delayed (>6 wks)	HC-HJ	Yes	Not significant
5	Omar et al., 2023 (RCT) [25]	277	Group A: ↑	Group A (no sepsis ctrl): ↓ success	HJ only	Yes (10 centers)	Sepsis ↑ complications
6	Nawacki et al., 2023 [2]	22	22.7%	Mostly early (<2 weeks)	63.6% HJ	Single tertiary	Yes, in emergency LC
7	Alvear-Torres et al., 2022 [33]	70	2.9%	Both early and late	78.3% HJ	Yes	12.9% vascular injury
8	Bobkiewicz et al., 2014 [34]	69	4.3%	Mixed	Mostly HJ + ERCP	Yes	Septic shock caused death
9	Anand et al., 2021 [27]	105	2.8%	Delayed repair common	100% HJ	Yes	Postop cholangitis ↑ risk
10	Schreuder et al., 2020 (MA) [14]	2484	NS	No mortality difference by timing	HJ mostly	Multicenter	NS

↑ indicates increased mortality risk or higher observed mortality. ↓ indicates decreased mortality risk or lower observed mortality.

**Table 12:** GRADE Summary of Certainty of Evidence for Primary Outcomes.

Outcome	Certainty	Reason for Rating
Surgical success (HJ)	High ⬤⬤⬤⬤	Consistent results, low risk of bias, precise effect estimates, low heterogeneity
Anastomotic stricture	Moderate ⬤⬤⬤◯	Moderate heterogeneity, varied definitions, minor imprecision
Postoperative complication rate	Moderate ⬤⬤⬤◯	High heterogeneity, variable timing, but consistent trend
Postoperative mortality	Low ⬤⬤◯◯	Wide confidence intervals, high heterogeneity, possible reporting bias

⬤ = filled circle indicating a point of certainty; ◯ = empty circle indicating reduced certainty. The number of filled versus empty circles represents the overall GRADE certainty level (from high ⬤⬤⬤⬤ to very low ⬤◯◯◯).

## Data Availability

No new data were created or analyzed in this study. Data supporting the findings of this review are available in the cited publications and publicly accessible databases (PubMed and the Cochrane Library).

## Supplementary Materials

The following supporting information can be downloaded at: https://www.mdpi.com/article/10.3390/life15121858/s1.

## Abbreviations

The following abbreviations are used in this manuscript:

Abbreviation	Definition
IBDI	Iatrogenic bile duct injury
BDI	Bile duct injury
LC	Laparoscopic cholecystectomy
OC	Open cholecystectomy
CVS	Critical view of safety
IOC	Intraoperative cholangiography
LUS	Laparoscopic ultrasound
NIRF-C	Near-infrared fluorescence cholangiography
HJ	Hepaticojejunostomy
HC-HJ	Hepp–Couinaud hepaticojejunostomy
ERCP	Endoscopic retrograde cholangiopancreatography
MRCP	Magnetic resonance cholangiopancreatography
PTC	Percutaneous transhepatic cholangiography
PTBD	Percutaneous transhepatic biliary drainage
CT	Computed tomography
MRI	Magnetic resonance imaging
3D	Three-dimensional
CHD	Common hepatic duct
CBD	Common bile duct
DBS	Delayed biliary stricture
SE	Standard error
SD	Standard deviation
CI	Confidence interval
OR	Odds ratio
RR	Risk ratio
MA	Meta-analysis
RCT	Randomized controlled trial
RoB 2	Revised Cochrane Risk of Bias Tool for Randomized Trials
NOS	Newcastle–Ottawa Scale
GRADE	Grading of Recommendations Assessment, Development and Evaluation
I^2^	I-squared (measure of heterogeneity)
τ^2^/Tau^2^	Tau-squared (between-study variance)
Q	Cochran’s Q statistic
PRISMA	Preferred Reporting Items for Systematic Reviews and Meta-Analyses
MOOSE	Meta-analysis of Observational Studies in Epidemiology
PROSPERO	International Prospective Register of Systematic Reviews
ASA	American Society of Anesthesiologists
NSQIP	National Surgical Quality Improvement Program
MeSH	Medical Subject Headings
SPSS	Statistical Package for the Social Sciences
RevMan	Review Manager (Cochrane Collaboration software)
NIH	National Institutes of Health
HPB	Hepatopancreatobiliary
FMS	Full mucosal sleeve
Spec	Specialist (e.g., HPB specialist)
No.	Number
n	Number (sample size)
w/	With
w/o	Without
