# Supplementary material for: Management of Iatrogenic Bile-Duct Injury After Cholecystectomy, 1995–2025: Systematic Review and Meta-Analysis
Source: Life (Basel). 2025 Dec 3;15(12):1858. doi: 10.3390/life15121858 (PMC12734171; doi:10.3390/life15121858)
Supplement: Supplementary file 1 [file life-15-01858-s001.zip › life-3985344-supplementary.pdf]

Supplementary Material

Search Terms (1990-2025)

"Iatrogenic bile duct injury" OR "iatrogenic biliary injury" OR "bile duct injury" OR "biliary injury" OR "bile duct stricture" OR "biliary stricture" OR "bile duct leak"

"Management" OR "treatment" OR "endoscopic intervention” OR "ERCP" OR "stent" OR "percutaneous drainage" OR "surgical repair" OR "hepaticojejunostomy" OR “Roux-en-Y"

Abbreviation	Definition
IBDI	Iatrogenic bile duct injury
BDI	Bile duct injury
LC	Laparoscopic cholecystectomy
OC	Open cholecystectomy
CVS	Critical view of safety
IOC	Intraoperative cholangiography
LUS	Laparoscopic ultrasound
NIRF-C	Near-infrared fluorescence cholangiography
HJ	Hepaticojejunostomy
HC-HJ	Hepp–Couinaud hepaticojejunostomy
ERCP	Endoscopic retrograde cholangiopancreatography
MRCP	Magnetic resonance cholangiopancreatography
PTC	Percutaneous transhepatic cholangiography
PTBD	Percutaneous transhepatic biliary drainage
CT	Computed tomography
MRI	Magnetic resonance imaging
3D	Three-dimensional
CHD	Common hepatic duct
CBD	Common bile duct
DBS	Delayed biliary stricture

**SE**	Standard error
**SD**	Standard deviation
**CI**	Confidence interval
**OR**	Odds ratio
**RR**	Risk ratio
**MA**	Meta-analysis
**RCT**	Randomized controlled trial
**RoB 2**	Revised Cochrane Risk of Bias Tool for Randomized Trials
**NOS**	Newcastle–Ottawa Scale
**GRADE**	Grading of Recommendations Assessment, Development and Evaluation
**I^2**	I-squared (measure of heterogeneity)
**τ^2 / Tau^2**	Tau-squared (between-study variance)
**Q**	Cochran's Q statistic
**PRISMA**	Preferred Reporting Items for Systematic Reviews and Meta-Analyses
**MOOSE**	Meta-analysis of Observational Studies in Epidemiology
**PROSPERO**	International Prospective Register of Systematic Reviews
**ASA**	American Society of Anesthesiologists
**NSQIP**	National Surgical Quality Improvement Program
**MeSH**	Medical Subject Headings
**SPSS**	Statistical Package for the Social Sciences
**RevMan**	Review Manager (Cochrane Collaboration software)
**NIH**	National Institutes of Health
**HPB**	Hepatopancreatobiliary
**FMS**	Full mucosal sleeve
**Spec**	Specialist (e.g., HPB specialist)
**No.**	Number
**n**	Number (sample size)
**w/**	With
**w/o**	Without

Supplementary Table S1.

Table S1: Study Summary.

**Reference**	**Study Design**	**N (BDI)**	**Setting / Population**	**Intervention(s) / Comparator(s)**	**Injury Classification**	**Outcomes Measured**	**Key Findings (Condensed)**	**Risk of Bias**
Nawacki et al. (2022) [1]	Retrospective cohort	22	Single center, Poland (2000–2020)	Surgical repair (HJ/T-tube)	EAES	Complications, mortality, stricture	Early complications 50%, mortality 22.7%, stricture 4.5%; all managed in tertiary center	Moderate (NOS)
Hogan et al. (2016) [2]	Prospective cohort	78	National liver/HPB unit, Ireland (1992–2014)	Surgery/endoscopy by period	Strasberg	Complications, mortality, repair type	Increased complexity/vascular injury over time; 28% complication, 3.8% mortality, 59% single HJ	Good (NOS)
Pesce et al. (2019) [3]	Narrative review	—	Synthesis of primary studies	Review of modalities and strategies	Multiple	Imaging, repair, risk factors	Multimodal imaging and early referral to HPB center are crucial for optimal outcome	—
Symeonidis et al. (2023)[4]	Systematic review	—	PRISMA-based, 85 studies	BILE classification and treatment	BILE/Strasberg	Algorithm, treatment, risk factors	New "BILE" system, supports specialist referral, sepsis control, and multidisciplinary management	—
Yang FQ et al. (2002) [5]	Retrospective cohort	182	Major center, China	Surgery, drainage, endoscopy	Bismuth	Complications, mortality, stricture	Mortality 6%, stricture 11.6%; delayed referral and vascular injury predicted poor outcome	—
Törnqvist et al. (2009) [6]	Population registry	1,386	Sweden, national registry	Surgical repair (suture, HJ)	By repair	Mortality, stricture, hospital level	1-yr mortality 15.8%; IOC use protective, hospital level not predictive	High (NOS)

Halle-Smith et al. (2022) [7]	Retrospective cohort	139	UK specialist HPB center	Specialist vs. non-specialist repair	Strasberg E	Long-term complications, survival	Specialist repair reduces complications (HR 0.25); vascular injury, non-specialist repair increase risk	Good (NOS)
Schreuder et al. (2020a) [8]	Retrospective + review	836/91	Amsterdam UMC + literature	HJ, endoscopy, PTBD	Strasberg-Bismuth	Stricture, mortality, QoL	10–20% stricture, specialist repair lowers risk, late strictures common	Good (NOS)
Schreuder et al. (2020b) [9]	Systematic review/meta-an.	2,484	21 studies, multiple centers	HJ timing: early, intermediate, delayed	Varies	Morbidity, stricture, mortality	Intermediate timing (2–6w) = higher stricture/morbidity; no mortality difference; HPB repair recommended	Moderate (NOS)
Conde Monroy et al. (2022) [10]	Retrospective multicenter	44	4 hospitals, Colombia	Early (<72h) vs. delayed (>72h) repair	Strasberg E2–E4	Complications, stricture, mortality	16% stricture, 2.3% mortality, no difference by timing; all repairs by HPB surgeon	Good (NOS)
Renz et al. (2017) [11]	Narrative review	—	Literature review	Review surgical therapy and outcome	Strasberg/Bismuth/Stewart	Repair type, outcomes	Early referral, expert repair, and sepsis control critical; >90% success with specialist	—
Spiers et al. (2023) [12]	Systematic review	179	7 cohorts, patients needing LT for BDI	Liver transplantation	Strasberg E	Complications, mortality, retransplant	20% 30-day mortality, 46.5% major complications, 5-year survival ~72%	—
Shallaly & Cuschieri (2000) [13]	Narrative review/audit	—	Medline audit, focus on LC injuries	Registry and audit synthesis	Bismuth/Strasberg	Incidence, complications, outcomes	Early diagnosis/referral, specialist repair critical; vascular injury = worst prognosis	—

Kholdebarin et al. (2008) [14]	Case-control	28	18 hospitals, Canada	Analysis of risk factors for BDI	Not specified	Risk factors	Failure to identify cystic duct and emergency LC are strongest risk factors	Good (NOS)
Rystedt et al. (2016) [15]	Registry cohort	174	GallRiks, Sweden, national	Repair type and timing stratified	Hannover	Stricture, sequelae, mortality	18% stricture, 3.4% mortality, early specialist referral protective	High (NOS)
Al Tamimi (2019) [16]	RCT	2,240	Iraq, elective LC	Traction-release step vs standard	Strasberg	Major/minor BDI, CBD tenting	0% major BDI in intervention; step reduces injury risk	Moderate (RoB2)
Omar et al. (2023) [17]	RCT	277	Egypt, multicenter	Early HJ (± sepsis ctrl) vs delayed	Strasberg E1–E4	Success, complications, QoL, mortality	Sepsis control, not timing, predicts success (OR 2.98); early HJ w/ sepsis ctrl best outcomes	Low (RoB2)
Kambakamba et al. (2022) [18]	Systematic review	15,609	24 studies, post-LC BDI	Immediate, early, delayed, late repair	Strasberg/Bismuth/Stewart	Timing, complications, mortality	No timing effect; sepsis, complexity, expertise determine outcome; 28% morbidity	Moderate (NOS)
Ma et al. (2021) [19]	Retrospective + animal	18	Hubei, China + rats	Novel end-to-end FMS anastomosis	Strasberg-Bismuth E1–E4	Patency, stricture, cholangitis	0% stricture/cholangitis at 5y in humans; FMS superior to traditional in animals	Moderate (NOS)
Anand et al. (2021) [20]	Retrospective cohort	105	AIIMS, India, post-LC BDI	Roux-en-Y HJ for all	Strasberg-Bismuth I–IV	McDonald outcome, complications	91.5% good/excellent; 6.7% strictures; hilar strictures/cholangitis predicted failure	Good (NOS)
Giuliante et al. (2023) [21]	Retrospective cohort	114	Rome, Italy, HPB center	Hepp–Couinaud HJ, timing stratified	Strasberg E1–E5	Patency, stricture, bile leak, mortality	No mortality; 94.7% patency; bile leak only predictor of failure; timing not significant	High (NOS)

Machi et al. (2009) [22]	Multicenter retrospective	1,381	5 high-volume US centers	Routine LUS vs. selective IOC	Not specified	BDI, bile leak, conversion	0% major BDI; 0.2% bile leak; LUS success 98%; reduced need for IOC	Moderate (NOS)
Yang Z et al. (2024) [23]	Retrospective cohort	15	China, early repair post-LC	Early HJ with 3D imaging guidance	Strasberg-Bismuth C, E1–E4	Operative outcomes, complications	100% imaging accuracy; 1 bile leak; no strictures/stones in 12–56m f/u	Moderate (NOS)
Felekouras et al. (2015) [24]	Retrospective cohort	92	Athens, HPB center	Early (<2w) vs late (>12w) HJ	Strasberg A–E5	Morbidity, mortality, stricture	No difference by timing; specialist repair reduces stricture/redo surgery	High (NOS)
Mishra et al. (2015)[25]	Retrospective cohort	137	India, tertiary HPB center	Stratified repair; HJ main	Strasberg, Bismuth	Morbidity, mortality, outcome success	97% HJ success, 4.4% mortality, main risks: sepsis/portal HTN, delayed referral	Good (NOS)
Alvear-Torres (2022)[26]	Retrospective observational	70	Mexico, tertiary hospital	Surgery (78%), endoscopy (22%)	Strasberg-Bismuth A–E5	Morbidity, mortality, stricture	37% morbidity, 2.9% mortality, 9.6% strictures; timing of repair not predictive	Moderate (NOS)
Bobkiewicz et al. (2015)[27]	Retrospective cohort	69	Poland, HPB center	HJ (35%), end-to-end, ERCP	Bismuth/Neuhaus	Complications, mortality, timing	HJ most effective; 4.3% mortality; delayed/failed initial repair = poor outcomes	Moderate (NOS)
Halle-Smith et al. (2022b)[28]	Retrospective cohort	44	UK, HPB center, long-term f/u	Surgical (91% HJ), non-op in select	DBS (delayed stricture >28d)	Late complications, mortality	36% late biliary complications, 2% biliary mortality; most DBS needed HJ	Good (NOS)

MOOSE Checklist

**Item**	**Reported**	**Location in Manuscript**
**Background**		
Problem definition	Yes	Abstract, Introduction
Hypothesis statement	Yes	Abstract, Introduction
Study outcomes described	Yes	Methods, Results
Exposure/intervention described	Yes	Methods—Study Characteristics
Study design specified	Yes	Abstract, Methods
Study population defined	Yes	Methods—Eligibility Criteria
**Search Strategy**		
Searchers' qualifications	Yes	Methods—Data Extraction (two independent reviewers)
Full search strategy	Yes	Methods—Search Strategy
Comprehensive effort to include all studies	Yes	Methods—Contact with authors, multiple databases
Databases/registries listed	Yes	Methods—Search Strategy
Search software detailed	Yes	Methods—EndNote X9 used for reference management
Hand searching described	Yes	Methods—Manual search (Google Scholar)
Number of citations included/excluded	Yes	PRISMA Flowchart, Results
Non-English articles addressed	Yes	Methods—No language restrictions
Handling of abstracts/unpublished data	Yes	Methods—Exclusion criteria specified
Author contact for missing data	Yes	Methods—Contacted when feasible
**Methods**		
Study relevance justified	Yes	Methods—Eligibility, Discussion
Data selection and coding process	Yes	Methods—Data Extraction (two reviewers)
Data classification described	Yes	Methods—Structured forms, consensus process
Confounding assessed	Yes	Discussion—Sepsis, comorbidities, referral center
Study quality assessment	Yes	Methods, Results—NOS, RoB2
Heterogeneity evaluated	Yes	Methods, Results—I^2, τ^2, Q statistic
Statistical methods detailed	Yes	Methods—Meta-analysis, random-effects, subgroup
Tables and figures provided	Yes	Tables 1+, Forest and Funnel plots
**Results**		
Descriptive table for each study	Yes	Results—Table 1
Sensitivity analysis	Yes	Results—Subgroup and sensitivity analyses

Statistical uncertainty reported	Yes	Results—Confidence intervals, p-values
**Discussion**		
Quantitative bias assessment	Yes	Results—Funnel plot, I^2, τ^2
Rationale for study exclusions	Yes	Methods, PRISMA diagram
Included study quality discussed	Yes	Results—Quality of Included Studies
**Conclusions**		
Alternative explanations considered	Yes	Discussion—Referral timing, center expertise
Generalizability addressed	Yes	Conclusion
Recommendations for future research	Yes	Conclusion
Funding disclosed	Yes	End—Funding statement

PRISMA 2020 Checklist

**Section**	**Item**	**Reported**	**Location in Manuscript**
**Title**	Identified as systematic review/meta-analysis	Yes	Title
**Abstract**	Structured summary of objectives, methods, results	Yes	Abstract
**Introduction**	Rationale for review	Yes	Introduction
	Objectives clearly stated	Yes	Introduction (final paragraph)
**Methods**	Eligibility criteria	Yes	Methods – "Eligibility Criteria"
	Information sources	Yes	Methods – "Search Strategy"
	Full search strategy	Yes	Supplementary Materials
	Selection process (reviewers, criteria)	Yes	Methods – "Study Selection and Data Extraction"
	Data collection process	Yes	Methods – "Data Extraction"
	Data items (outcomes, covariates)	Yes	Methods – "Outcomes", Table 1
	Risk of bias assessment	Yes	Methods – "Assessment of Methodological Quality"
	Effect measures	Yes	Methods – "Data Synthesis and Analysis"
	Synthesis methods	Yes	Methods – "Meta-analysis", Heterogeneity (I^2, τ^2)
	Reporting bias assessment	Yes	Methods/Results – "Funnel Plot"
	Certainty of evidence (GRADE)	Yes	Methods/Results – "Certainty of Evidence (GRADE Assessment)"
**Results**	Study selection + PRISMA diagram	Yes	Results – "Search Results", Figure 1
	Study characteristics	Yes	Results – "Characteristics of Included Studies"
	Risk of bias in included studies	Yes	Results – "Quality of Included Studies"
	Results of individual studies	Yes	Table 1, Forest Plots
	Results of syntheses	Yes	Results – Meta-analysis
	Reporting biases	Yes	Results – Funnel Plot

	Certainty of evidence	Yes	Results – GRADE Table
**Discussion**	Interpretation of results	Yes	Discussion
	Limitations of evidence	Yes	Discussion – Bias, Heterogeneity
	Conclusions	Yes	Conclusion section
**Other Info**	Registration & protocol (e.g., PROSPERO)	Yes	Methods – PROSPERO CRD420251003227
	Support/funding	Yes	Funding – "No support received"
	Competing interests	Yes	Competing Interests
	Data/materials availability	Yes	Data Availability Statement

1. Nawacki Ł, Kozłowska-Geller M, Wawszczak-Kasza M, Klusek J, Znamirowski P, Głuszek S. Iatrogenic Injury of Biliary Tree-Single-Centre Experience. Int J Environ Res Public Health. 2022;20(1).
2. Hogan NM, Dorcaratto D, Hogan AM, Nasirawan F, McEntee P, Maguire D, Geoghegan J, Traynor O, Winter DC, Hoti E. Iatrogenic common bile duct injuries: Increasing complexity in the laparoscopic era: A prospective cohort study. Int J Surg. 2016;33 Pt A:151-6.
3. Pesce A, Palmucci S, La Greca G, Puleo S. Iatrogenic bile duct injury: impact and management challenges. Clin Exp Gastroenterol. 2019;12:121-8.
4. Symeonidis D, Tepetes K, Tzovaras G, Samara AA, Zacharoulis D. BILE: A literature review based novel clinical classification and treatment algorithm of iatrogenic bile duct injuries. Journal of Clinical Medicine. 2023;12(11):3786.
5. Yang FQ, Dai XW, Wang L, Yu Y. Iatrogenic extrahepatic bile duct injury in 182 patients: causes and management. Hepatobiliary Pancreat Dis Int. 2002;1(2):265-9.
6. Törnqvist B, Zheng Z, Ye W, Waage A, Nilsson M. Long-term effects of iatrogenic bile duct injury during cholecystectomy. Clin Gastroenterol Hepatol. 2009;7(9):1013-8; quiz 915.
7. Halle-Smith JM, Marudanayagam R, Mirza DF, Roberts KJ. Long-term outcomes of delayed biliary strictures following cholecystectomy. HPB (Oxford). 2022;24(2):209-16.
8. Schreuder AM, Busch OR, Besselink MG, Ignatavicius P, Gulbinas A, Barauskas G, Gouma DJ, van Gulik TM. Long-Term Impact of Iatrogenic Bile Duct Injury. Dig Surg. 2020;37(1):10-21.
9. Schreuder AM, Nunez Vas BC, Booij KAC, van Dieren S, Besselink MG, Busch OR, van Gulik TM. Optimal timing for surgical reconstruction of bile duct injury: meta-analysis. BJS Open. 2020;4(5):776-86.
10. Conde Monroy D, Torres Gómez P, Rey Chaves CE, Recamán A, Pardo M, Sabogal JC. Early versus delayed reconstruction for bile duct injury a multicenter retrospective analysis of a hepatopancreaticobiliary group. Scientific Reports. 2022;12(1):11609.
11. Renz BW, Bösch F, Angele MK. Bile duct injury after cholecystectomy: surgical therapy. Visceral medicine. 2017;33(3):184-90.
12. Spiers HVM, Lam S, Machairas NA, Sotiropoulos GC, Praseedom RK, Balakrishnan A. Liver transplantation for iatrogenic bile duct injury: a systematic review. HPB (Oxford). 2023;25(12):1475-81.
13. Shallaly G, Cuschieri A. Nature, aetiology and outcome of bile duct injuries after laparoscopic cholecystectomy. HPB. 2000;2(1):3-12.

14. Kholdebarin R, Boetto J, Harnish JL, Urbach DR. Risk factors for bile duct injury during laparoscopic cholecystectomy: a case-control study. Surgical innovation. 2008;15(2):114-9.
15. Rystedt J, Lindell G, Montgomery A. Bile Duct Injuries Associated With 55,134 Cholecystectomies: Treatment and Outcome from a National Perspective. World J Surg. 2016;40(1):73-80.
16. Al Tamimi AS. An Additional Step in the Technique of Laparoscopic Cholecystectomy can Minimize the Incidence of Major Bile Duct Injury. Journal of Pharmaceutical Sciences and Research. 2019;11(1):159-62.
17. Omar MA, Kamal A, Redwan AA, Alansary MN, Ahmed EA. Post-cholecystectomy major bile duct injury: ideal time to repair based on a multicentre randomized controlled trial with promising results. Int J Surg. 2023;109(5):1208-21.
18. Kambakamba P, Cremen S, Möckli B, Linecker M. Timing of surgical repair of bile duct injuries after laparoscopic cholecystectomy: A systematic review. World J Hepatol. 2022;14(2):442-55.
19. Ma D, Liu P, Lan J, Chen B, Gu Y, Li Y, Yue P, Liu Z, Guo D. A Novel End-to-End Biliary-to-Biliary Anastomosis Technique for Iatrogenic Bile Duct Injury of Strasberg-Bismuth E1-4 Treatment: A Retrospective Study and in vivo Assessment. Front Surg. 2021;8:747304.
20. Anand U, Kumar R, Priyadarshi RN, Kumar M, Kumar R, Ahmed N, John AG, Parasar K, Kumar B. Analysis of outcomes of biliary reconstruction after post-cholecystectomy bile duct injuries. ANZ J Surg. 2021;91(7-8):1542-8.
21. Giuliante F, Panettieri E, De Rose AM, Murazio M, Vellone M, Mele C, Clemente G, Giovannini I, Nuzzo G, Ardito F. Bile duct injury after cholecystectomy: timing of surgical repair should be based on clinical presentation. The experience of a tertiary referral center with Hepp-Couinaud hepatico-jejunostomy. Updates Surg. 2023;75(6):1509-17.
22. Machi J, Johnson JO, Deziel DJ, Soper NJ, Berber E, Siperstein A, Hata M, Patel A, Singh K, Arregui ME. The routine use of laparoscopic ultrasound decreases bile duct injury: a multicenter study. Surg Endosc. 2009;23(2):384-8.
23. Yang Z, Liu J, Wu L, Ding Y, Ma S, Yan W, Lan Y, Sha X, Cheng J, Ma Z, Li M. Application of three-dimensional visualization technology in early surgical repair of bile duct injury during laparoscopic cholecystectomy. BMC Surgery. 2024;24(1):271.
24. Felekouras E, Petrou A, Neofytou K, Moris D, Dimitrokallis N, Bramis K, Griniatsos J, Pikoulis E, Diamantis T. Early or Delayed Intervention for Bile Duct Injuries following Laparoscopic Cholecystectomy? A Dilemma Looking for an Answer. Gastroenterol Res Pract. 2015;2015:104235.
25. Mishra PK, Saluja SS, Nayeem M, Sharma BC, Patil N. Bile Duct Injury-from Injury to Repair: an Analysis of Management and Outcome. Indian J Surg. 2015;77(Suppl 2):536-42.
26. Alvear-Torres LE, Estrada-Castellanos A. Lesión de vía biliar, experiencia de 3 años en un hospital de tercer nivel. Cirugía y cirujanos. 2022;90(4):508-16.
27. Bobkiewicz A, Krokowicz Ł, Banasiewicz T, Kościński T, Borejsza-Wysocki M, Ledwosiński W, Drews M. Iatrogenic bile duct injury. A significant surgical problem. Assessment of treatment outcomes in the department's own material. Pol Przegl Chir. 2015;86(12):576-83.
28. Halle-Smith JM, Hodson J, Stevens LG, Dasari B, Marudanayagam R, Perera T, Sutcliffe RP, Muiesan P, Isaac J, Mirza DF, Roberts KJ. A comprehensive evaluation of the long-term clinical and economic impact of minor bile duct injury. Surgery. 2020;167(6):942-9.
